# Epigallocatechin Gallate During Dietary Restriction — Potential Mechanisms of Enhanced Liver Injury

**DOI:** 10.3389/fphar.2020.609378

**Published:** 2021-01-29

**Authors:** Zhuo Shi, Jing-xiao Zhu, Yu-ming Guo, Ming Niu, Le Zhang, Can Tu, Ying Huang, Peng-yan Li, Xu Zhao, Zi-teng Zhang, Zhao-fang Bai, Guang-qin Zhang, Yang Lu, Xiao-he Xiao, Jia-bo Wang

**Affiliations:** ^1^ School of Basic Medicine and Clinical Pharmacy, China Pharmaceutical University, Nanjing, China; ^2^ China Military Institute of Chinese Medicine, Fifth Medical Center of Chinese PLA General Hospital, Beijing, China; ^3^ Hunan University of Chinese Medicine, Changsha, China; ^4^ College of Pharmacy, Southwest Minzu University, Chengdu, China; ^5^ Beijing Research Institute of Chinese Medicine, Beijing University of Chinese Medicine, Beijing, China; ^6^ Integrative Medical Center, Fifth Medical Center of Chinese PLA General Hospital, Beijing, China; ^7^ School of Traditional Chinese Medicine, Capital Medical University, Beijing, China

**Keywords:** green tea extract, epigallocatechin gallate, hepatotoxicity, combination effect, lipid metabolism, metabolomics, herbal and dietary supplements

## Abstract

Green tea extract (GTE) is popular in weight loss, and epigallocatechin gallate (EGCG) is considered as the main active component. However, GTE is the primary cause of herbal and dietary supplement-induced liver injury in the United States. Whether there is a greater risk of liver injury when EGCG is consumed during dieting for weight loss has not been previously reported. This study found for the first time that EGCG could induce enhanced lipid metabolism pathways, suggesting that EGCG had the so-called “fat burning” effect, although EGCG did not cause liver injury at doses of 400 or 800 mg/kg in normal mice. Intriguingly, we found that EGCG caused dose-dependent hepatotoxicity on mice under dietary restriction, suggesting the potential combination effects of dietary restriction and EGCG. The combination effect between EGCG and dietary restriction led to overactivation of linoleic acid and arachidonic acid oxidation pathways, significantly increasing the accumulation of pro-inflammatory lipid metabolites and thus mediating liver injury. We also found that the disruption of Lands’ cycle and sphingomyelin-ceramides cycle and the high expression of taurine-conjugated bile acids were important metabolomic characteristics in EGCG-induced liver injury under dietary restriction. This original discovery suggests that people should not go on a diet while consuming EGCG for weight loss; otherwise the risk of liver injury will be significantly increased. This discovery provides new evidence for understanding the “drug-host” interaction hypothesis of drug hepatotoxicity and provides experimental reference for clinical safe use of green tea-related dietary supplements.

## Introduction

Green tea is a beverage that has been brewed for thousands of years and has been widely popular across the world for hundreds of years. It is rich in catechin compounds with antioxidant effect, and epigallocatechin gallate (EGCG) is the most important component of green tea extract (GTE). It is popularly believed to have a wide range of health benefits, including preventing cancer, lowering cholesterol, reducing inflammation, and delaying aging (Li et al., 2019). In recent years, GTE and its principal component EGCG have been reported to have the so-called “fat-burning” weight loss effect (Chen et al., 2016), which makes it one of the most popular herbal and dietary supplement (HDS) in the world. However, in contrast to these health benefits, the number of drug-induced liver injury (DILI) cases when GTE is used for weight loss has consistently increased, with GTE being the leading cause of HDS-DILI in the United States (Navarro et al., 2017). GTE accounts for more than 50% of the suspected HDS products that cause DILI (Navarro et al., 2013). In severe cases, there is risk of acute liver failure or mortality. According to clinical observational research results, individuals who fasted or lost significant weight seemed to be more prone to liver injury, or liver injury was more severe when consuming GTE (Pillukat et al., 2014; Zheng et al., 2016). Although the hepatotoxicity of GTE and its main component EGCG has been reported (Emoto et al., 2014; Wang et al., 2015), it is not clear why there are more reports of liver injury when GTE is used for weight loss.

The “fat-burning” functions of EGCG were considered to include promoting energy consumption and fatty acid oxidation, inhibiting fatty acid synthesis, and reducing nutrition absorption (Wolfram et al., 2006; Rains et al., 2011; Yamashita et al., 2018). Some studies have shown that the process of weight loss through dieting (calorie restriction) is also accompanied by the remodeling of fatty acid metabolic pathways, such as decomposing more fatty acids to provide calories, thus achieving the effect of fat loss (Bruss et al., 2010; Lee et al., 2015; Collet et al., 2017; Green et al., 2019). Dieting while consuming EGCG can enhance the catabolism of fatty acids, which may increase the effect of fat loss. However, there is no experimental evidence as to whether this superposition of fatty acid metabolism regulation may be involved in the occurrence process of EGCG liver injury, thereby increasing the risk of EGCG-induced liver injury. In this regard, we try to characterize the metabolic alterations induced by EGCG and the dietary restriction in mice plasma using metabolomics approach and investigate the potential liver injury due to EGCG and dietary restriction alone or in combination, so as to provide experimental reference for rational use of GTE products for weight loss.

## Materials and Methods

### Animal Grouping and Administration

Healthy female C57BL/6J mice, SPF grade, weighing 22–24 g, were purchased from SPF Experimental Technology Co., Ltd (License No.: SCXK (BJ) 2018-0010). Mice were provided with food and water and kept at 25 ± 2°C, 50–60% humidity, with 12 h/12 h light and dark cycles, in separate cages in the experimental animal center of the Fifth Medical Center of the Chinese PLA General Hospital. All animal experiments were approved by the Center for Laboratory Animal Welfare and Ethics of the Fifth Medical Center of the Chinese PLA General Hospital and followed the Animal Research Guidelines of National Institutes of Health. Epigallocatechin gallate (EGCG, purity ≥ 98%) was purchased from Chengdu Preferred Biotech Co., Ltd.

The mice were adaptively fed for 7 days, during which their food intake was monitored to obtain the average daily food intake per cage. The basic feed composition has crude protein (≥180 g/kg), crude fat (≥40 g/kg), total amino acids (≥76.6 g/kg), and various vitamins and micronutrients. The mice were then fed in the following two ways at 17:00 every day for 6 days: 1) free diet and 2) fixed diet, that is, 50% of the average daily food intake (limited less than 2 g each mouse 1 day). On the sixth day, the mice on the free diet were randomly divided into a control group (control), low-dose EGCG group (400 mg/kg), and high-dose EGCG group (800 mg/kg). Meanwhile, the mice on the fixed diet were randomly divided into three groups: dieting group, dieting/low-dose EGCG group (400 mg/kg), and dieting/high-dose EGCG group (800 mg/kg). The four administration groups were treated with intragastric EGCG of the corresponding dose at the same time. The control group and the dieting group were given equal volume of normal saline through intragastric means. There were eight mice in each group. After 24 h, the animals were sacrificed, blood was collected, and plasma was centrifuged at 4°C and frozen at −80°C. Fresh mice liver tissues were washed with normal saline and soaked in 4% neutral paraformaldehyde solution.

### Evaluation of Blood Biochemistry and Liver Histology

The frozen plasma was returned to room temperature, and alanine aminotransferase (ALT) and aspartate aminotransferase (AST), glutathione S-transferase (GST) activity, and total bile acid (TBA) concentration were measured and calculated according to the kit instructions. ALT kit (No.: C009-2), AST kit (No.: C010-2), GST kit (No.: A004), and TBA kit (No.: E003-2-1) were purchased from Nanjing Jiancheng Bioengineering Institute.

The liver tissues of mice were collected, and routine histological sections were stained with hematoxylin and eosin (HE). Then, the pathological sections were photographed under a microscope. Terminal deoxynucleotidyl transferase dUTP nick end labeling (TUNEL) was used to detect and analyze the apoptosis of hepatocytes. Image-Pro Plus 6.0 software was used to label and calculate the apoptosis rate.

### Metabolomics Analysis

We selected plasma from the control, dieting, high-dose EGCG (800 mg/kg), and dieting/high-dose EGCG (800 mg/kg) groups as metabolomics test samples. The mice samples stored at −80°C refrigeration were thawed to 4°C, and 50 μl of each sample was pipetted into the 1.5 ml EP tube; three times the volume of precooled methanol was added, vortexed for 30 s, and kept standing at 4°C for 30 min. It was then centrifuged at 14,000 rpm for 15 min in a precooled 4°C centrifuge; 100 μl of supernatant was pipetted and dried with nitrogen. 75% methanol of the same volume was added to reconstitute the sample to be tested. Meanwhile, 10 μl of each sample was taken and mixed evenly and used as the quality control (QC) sample. For plasma metabolite chromatographic separation conditions and non-targeted metabolomics mass spectrometry detection conditions, please see the Supplementary Material.

Using MassHunter Qualitative Analysis software (version B06.00, Agilent, United States), LC-MS raw data was converted into visual data. MZmine software (version 2.5) was used for peak extraction, data correction, chromatographic deconvolution, and comparison. The raw data was displayed on the MetaboAnalyst webpage (https://www.metaboanalyst.ca/) and normalized and compared between groups, and metabolites with group differences were screened by calculating the fold change (FC) values and significant differences (*p* value) between groups. The criteria for differences between groups were as follows: FC was greater than 1.5 or less than 0.67, and *p* value was less than 0.05. At the same time, the obtained data matrix was imported into SIMCA-P software (version 14.1, Umetrics AB, Umea, Sweden) for principal component analysis (PCA) and orthogonal partial least squares discriminant analysis (OPLS-DA). Under the OPLS-DA model, the important variable (VIP) > 0.5 and |*p* (corr) |≥0.5 were used as the screening criteria for metabolites with inter-group differences. These metabolites were then identified using the Metlin database (http://www.metlin.scipps.edu/) and HMDB (http://www.hmdb.ca/) (mass error limited to 30 ppm). Pathway analysis based on the identified metabolites was conducted using MetaboAnalyst according to the Kyoto Encyclopedia of Genes and Genomes (KEGG) pathway database (www.genome.jp/kegg/). MS/MS spectra of mainly representative metabolites were in Supplementary Figure S4.

### Statistical Analysis

The experimental data was expressed as mean ± SD. GraphPad Prism software version 8 was used for statistical analysis, Student’s *t*-test was used for inter-group comparison, and analysis of variance (ANOVA) was used for one-way ANOVA. *p* < 0.05, *p* < 0.01, and *p* < 0.001 were considered to be statistically significant.

## Results

### EGCG Simulates Dieting Effects for Lipid Metabolism

**Figure 1:**
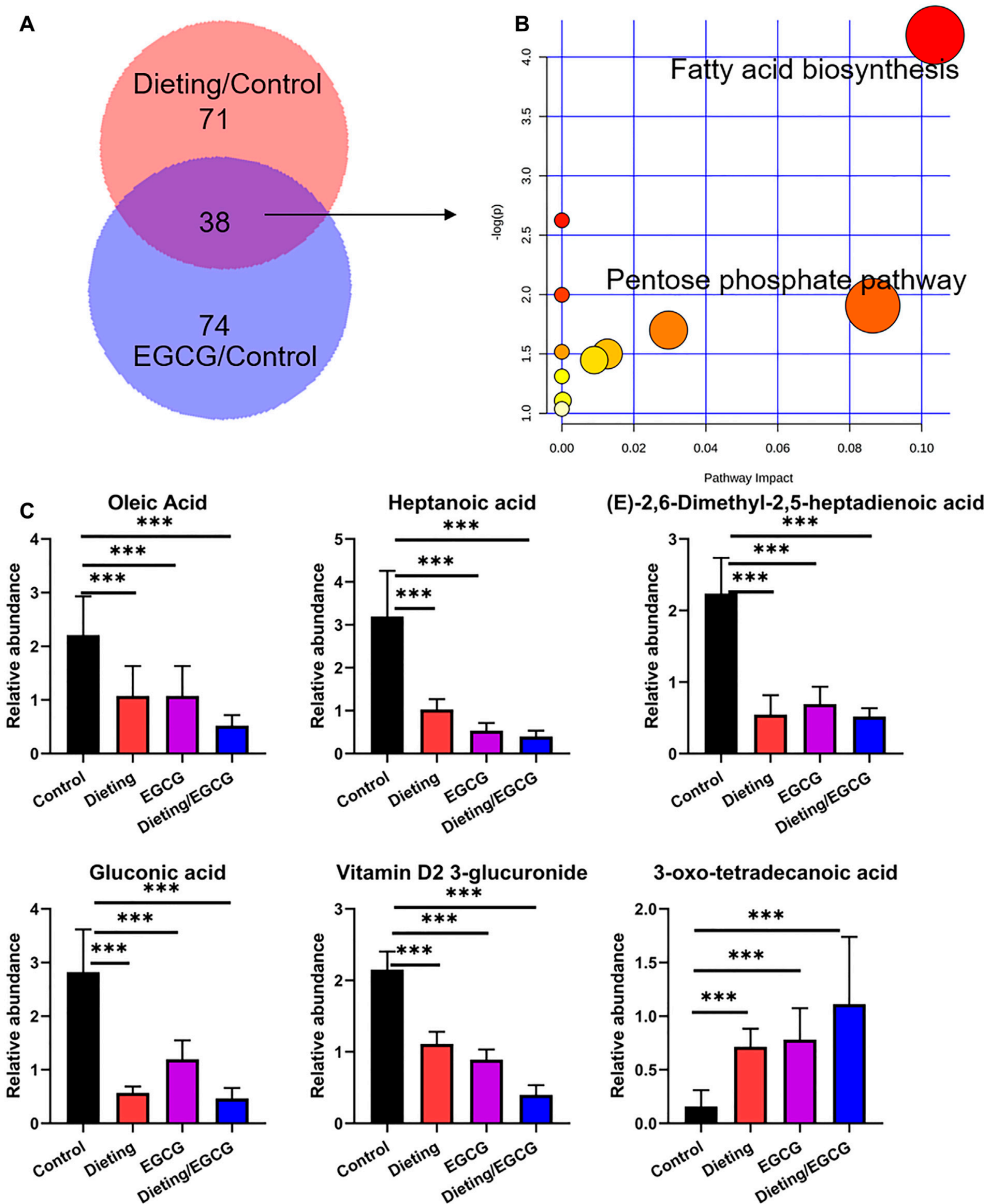
EGCG simulates the effect of dieting on fat loss. **(A)** The differential metabolites amount between dieting and control is displayed as pink, while that between EGCG and control is displayed as purple. The Venn diagram shows the shared metabolites between EGCG and dieting. **(B)** Pathway enrichment of 38 common metabolites. **(C)** Relative abundance of fat loss biomarkers. ***p < 0.001.

We screened and identified differential metabolites to study the metabolic changes caused by EGCG and diet on the mice. EGCG and diet coregulated metabolites as shown in the Venn diagram in Figure 1A. The pathways altered by EGCG and dieting mainly focused on fatty acid biosynthesis and pentose phosphate pathway (Figure 1B). Biomarkers included gluconic acid, oleic acid, heptanoic acid, (E)-2, 6-dimethyl-2, 5-heptadienoic acid, vitamin D2 3-glucuronide, and 3-oxo-tetradecanoic acid. The corresponding abundance changes were shown in Figure 1C, and the specific identification information was shown in Supplementary Table S1. We found that dietary restriction could slow down fatty acid synthesis, which could explain the fat loss effect caused by dieting. EGCG could also slow down fatty acid synthesis, which was consistent with its claimed weight loss effect. At the same time, we found that, in the dieting/EGCG group, the content of related biomarkers decreased further, but the excessive metabolic changes brought about by this combination may also cause adverse results. Therefore, we compared the liver injury induced by EGCG with that induced by EGCG under dietary restriction *in vitro* and *in vivo*.

**GRAPHICAL ABSTARCT:**
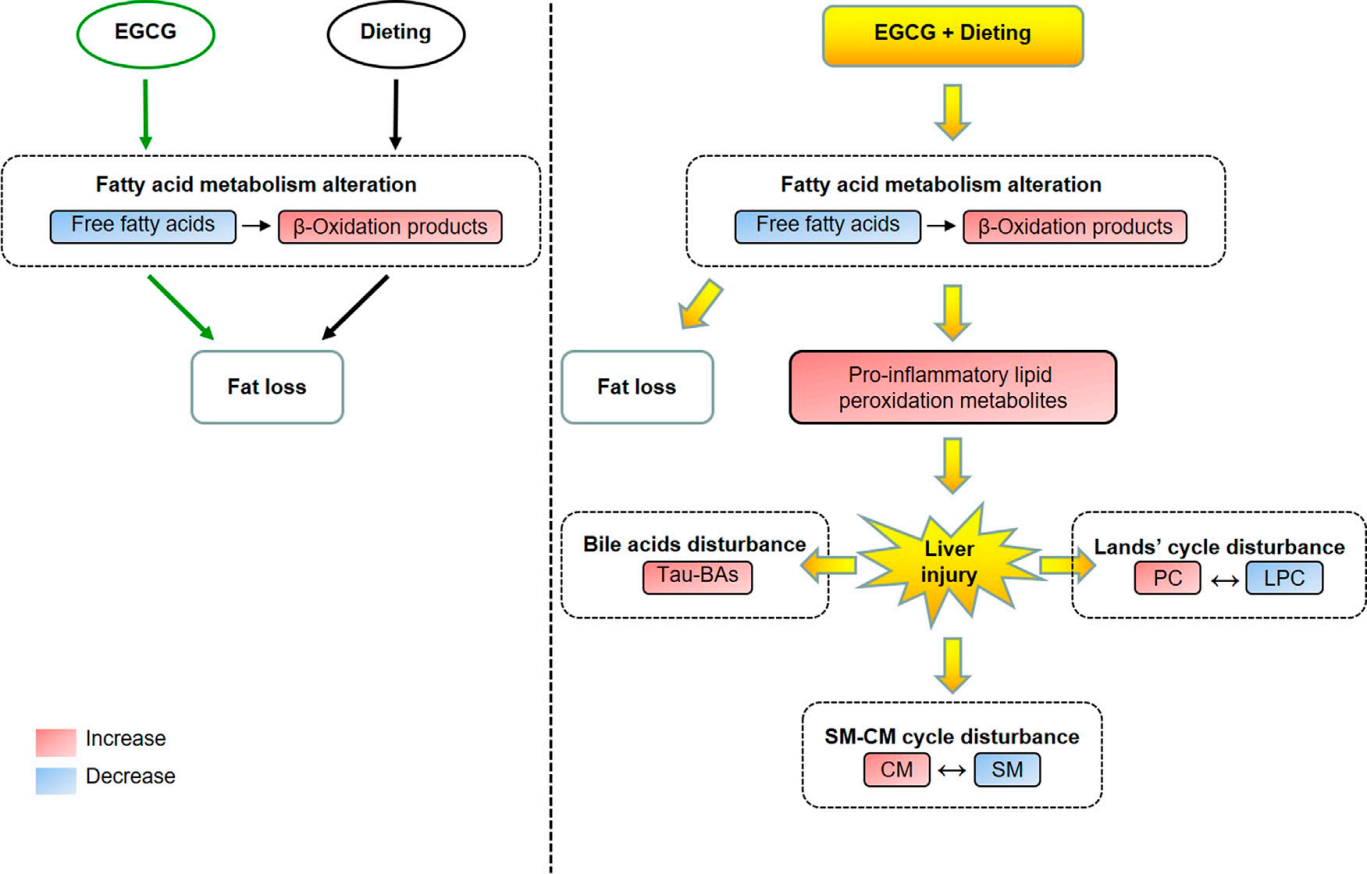
The metabolic alteration induced by EGCG in normal state and the metabolic homeostasis disturbance induced by EGCG during dieting. Tau-BAs, taurine-conjugated bile acids; PC, phosphatidylcholine; LPC, lysophosphatidylcholine; CM, ceramides; SM, sphingomyelin.

### Dietary Restriction Exacerbates EGCG Hepatotoxicity *In Vivo*

**Figure 2:**
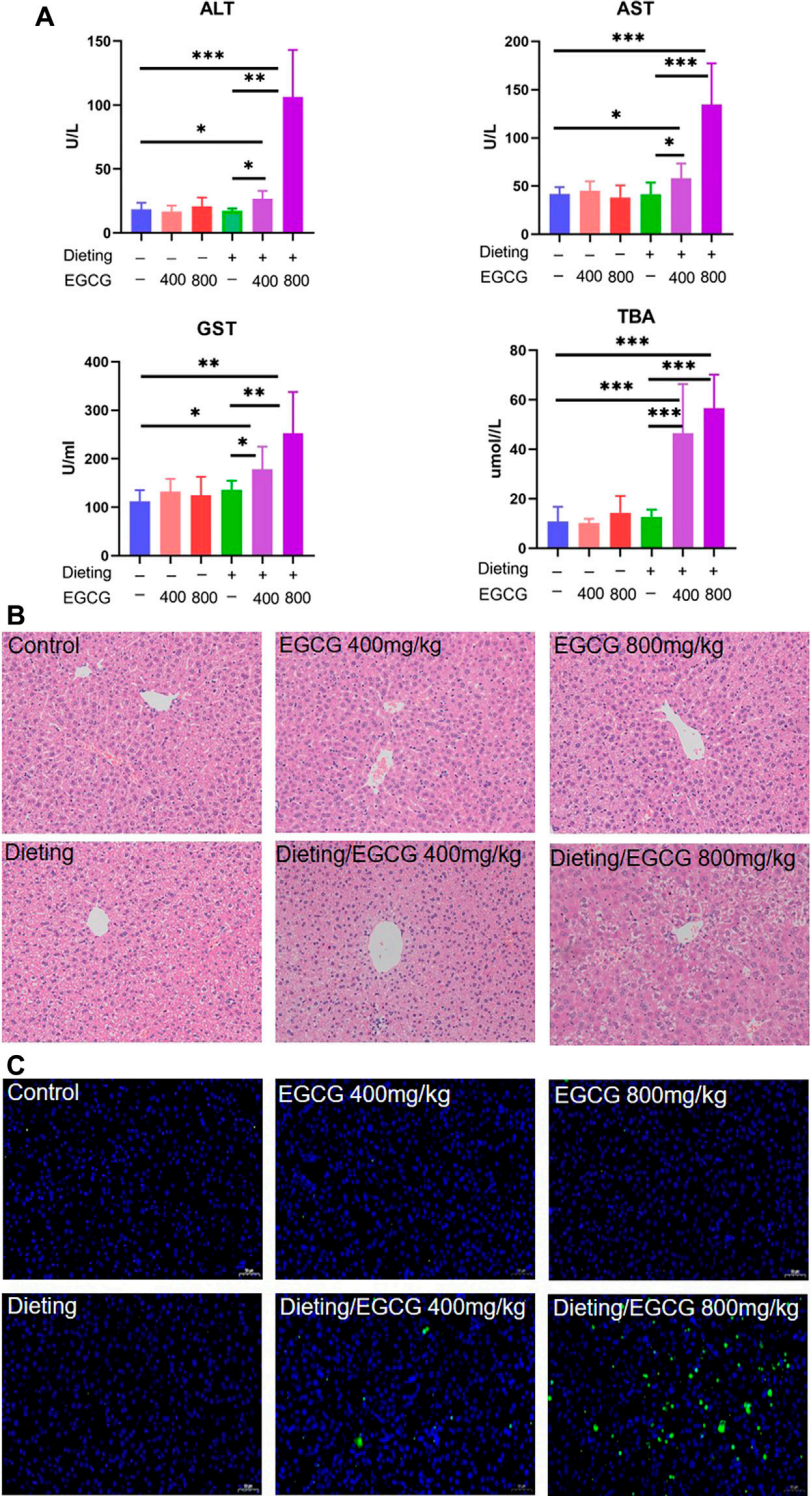
Dieting exacerbates EGCG hepatotoxicity in mice. **(A)** Plasma ALT; AST; GST; TBA. The data were expressed as mean ± SD. **(B)** Histological assessment of mice liver (HE staining ×200 magnification). **(C)** Hepatocyte apoptosis determination in mice liver using TUNEL assay (×200 magnification). *p < 0.05, **p < 0.01, and ***p < 0.001.

The experimental animal results (Figure 2A) showed that, compared with the control group, there were no statistical differences in biochemical indexes between dieting group and each EGCG single administration group (*p* > 0.05). However, there were significant increases on ALT, AST, GST, and TBA in the combination groups of dieting with EGCG (*p* < 0.01). The liver injury indicators in the high-dose (800 mg/kg) EGCG with dieting group were higher than those of the low-dose combination group (400 mg/kg).

The results of HE staining (Figure 2B) showed that the hepatocytes in the control, EGCG, and dieting groups represented with normal morphology. By contrast, obvious histological changes occurred in the diet combined with EGCG treatment group, including a decrease in the number of hepatocytes and some vacuolar degeneration, accompanied by infiltration of inflammatory cells. In the TUNEL staining results (Figure 2C), there were basically no green fluorescence positive cells in the visual field of the single administration group compared with the control group, and there was no statistical difference in the calculated apoptosis rate. Under dietary restriction conditions, green fluorescence positive cells were scattered in the visual field of 400 mg/kg EGCG administration group, and the apoptosis rate was significantly different from that of the control group (*p* < 0.05). There was more hepatocyte apoptosis in the visual field of 800 mg/kg EGCG combined with diet, and the apoptosis rate was significantly different from that in the control group (*p* < 0.01) (Supplementary Figure S1). The above results indicate that EGCG can cause increased hepatocyte apoptosis under dietary restriction.

### Metabolomics Analysis of Dietary Restriction Exacerbating EGCG Hepatotoxicity

**Figure 3:**
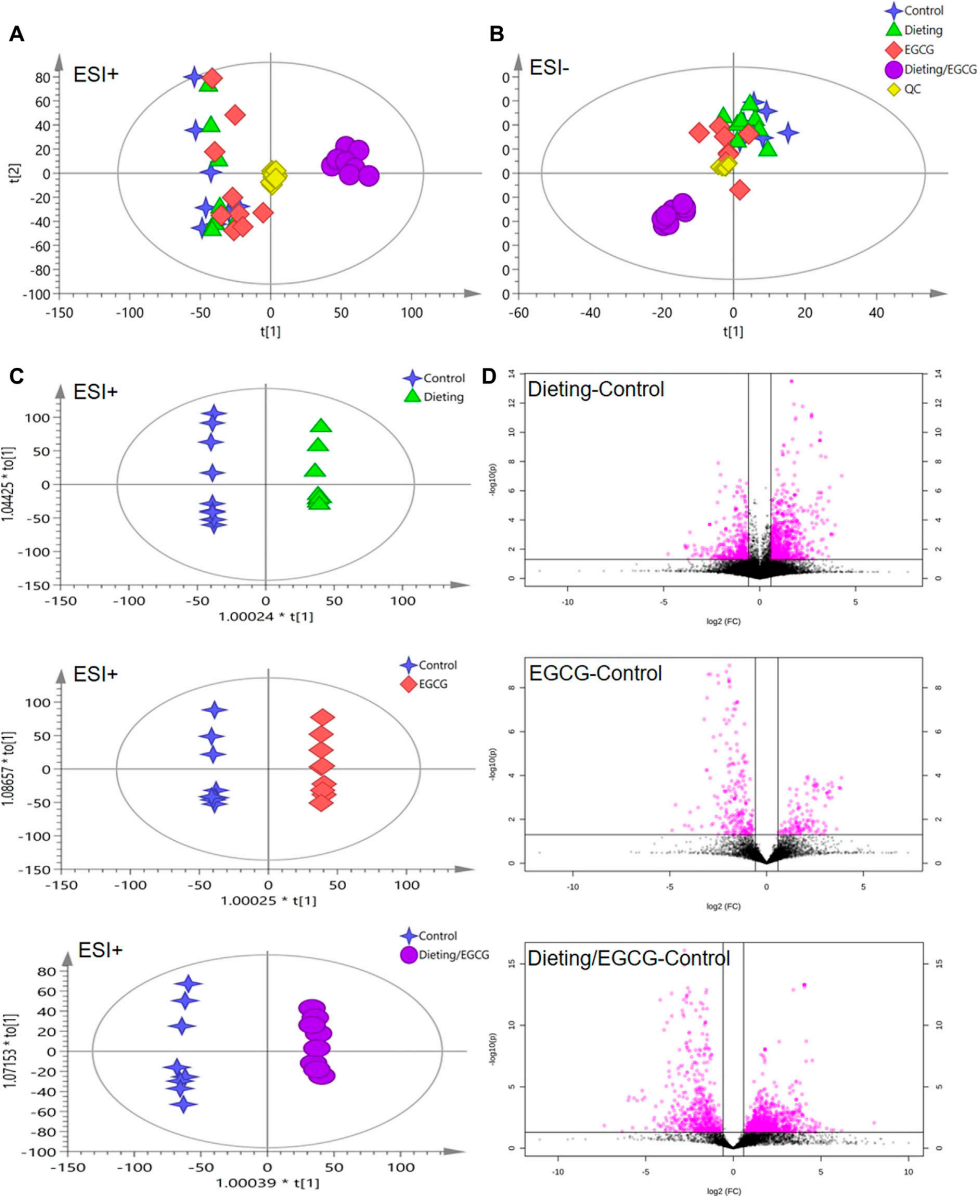
Metabolomic analysis of mice plasma. **(A)** The principal component analysis (PCA) score plot in positive ESI model. **(B)** The PCA score plot in negative ESI model. **(C)** The orthogonal projection to latent structures discriminant analysis (OPLS-DA) score plots in positive ESI model between dieting, EGCG, dieting/EGCG, and control. **(D)** The volcano plots in positive ESI model between dieting, EGCG, dieting/EGCG, and control.

PCA and OPLS-DA analysis were carried out on the data in positive and negative ion modes using SIMCA-P. Figures 3A,B displayed the scattered point plots of PCA of all samples in positive ion and negative ion modes. It could be seen that QC samples were concentrated in the middle of the score matrix projection plot, which indicated that the instrument remained stable during the analysis process. In addition, in positive ion mode, control, EGCG, and dieting groups were distributed on the left side of the first principal component, while dieting/EGCG group was located on the right side of the first principal component and was far away from control, EGCG, and dieting groups, suggesting that the metabolic profile of the dieting/EGCG group had obviously changed, which was consistent with the experimental phenotype of liver injury caused by EGCG under dietary restriction.

In order to further obtain the differences between groups and different compounds, OPLS-DA modeling and analysis were carried out. In the positive ion mode, dieting and control, EGCG and control, dieting/EGCG and control groups were located on both sides of the ordinate and were completely separated. Volcanic plots showed the metabolic changes in mice induced by dieting, EGCG, or dieting/EGCG (Figures 3C,D for positive ion and Supplementary Figure S2 for negative ion). Further, we used dieting/EGCG and control to screen the metabolic pathways related to liver injury. We found that some important pathways were alteration, including linoleic acid metabolism, glycerophospholipid metabolism, sphingolipid metabolism, taurine, and hypotaurine metabolism (Supplementary Figure S3).

### Dietary Restriction Exacerbating EGCG Hepatotoxicity Was Associated With Increased Production of Pro-Inflammatory Metabolites Through Arachidonic Acid and Linoleic Acid Metabolism

**Figure 4:**
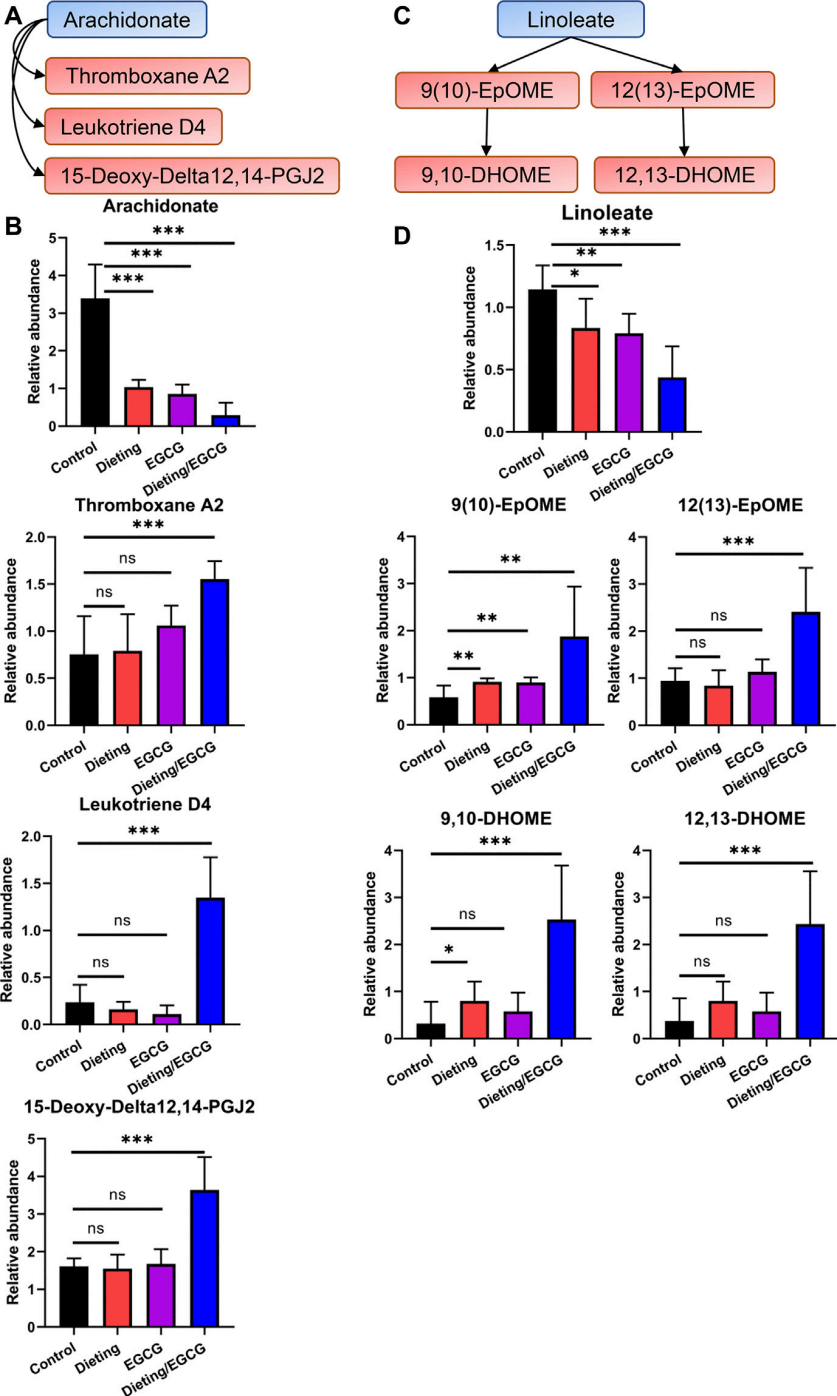
Dieting exacerbating EGCG hepatotoxicity promotes arachidonic acid and linoleate metabolism. **(A)** Alteration of arachidonic acid metabolism. Blue represents downregulation and red represents upregulation. **(B)** Relative abundance of arachidonate, 15-deoxy-delta12, 14-PGJ2, leukotriene D4, and thromboxane A2. **(C)** Alteration of linoleate metabolism. **(D)** Relative abundance of linoleate, 9 (10)-EpOME, 9,10-DHOME, 12 (13)-EpOME, 12,13-DHOME. *p < 0.05, **p < 0.01, and ***p < 0.001; ns, no significance.

Unsaturated fatty acid catabolism was significantly enhanced, which may be an important marker of lipid peroxidation; the plasma levels of arachidonic acid (Figure 4A) decreased significantly in the dieting/EGCG group, while the levels of its derived eicosanoid acids including 15-deoxy-delta12, 14-PGJ2, leukotriene D4, and thromboxane A2 significantly increased (Figure 4B). We also found that linoleic acid metabolism (Figure 4C) was disrupted, and the level of linoleate in dieting/EGCG group was significantly reduced. Its downstream metabolites including 9(10)-EpOME, 9,10-DHOME, 12(13)-EpOME, 12,13-DHOME showed different degrees of upregulation in the combined treatment group (Figure 4D). The above specific identification information is shown in Supplementary Tables S2, S3.

### Dietary Restriction Exacerbating EGCG Hepatotoxicity Disrupts Lands’ Cycle

**Figure 5:**
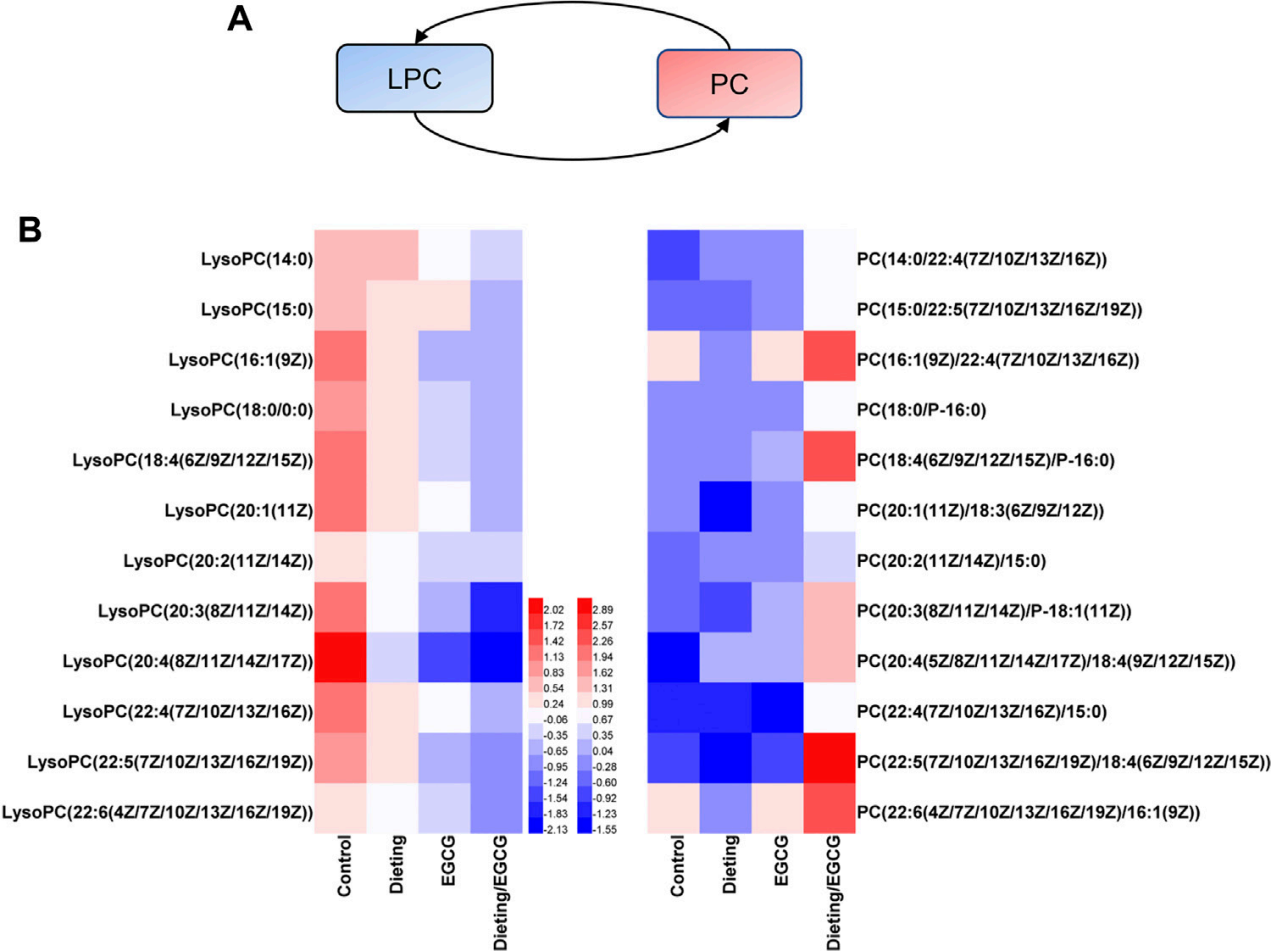
Dieting exacerbating EGCG hepatotoxicity disrupts Lands’ cycle. **(A)** Lands’ cycle. Blue represents downregulation and red represents upregulation. **(B)** Heatmap analysis of the identified Lands’ cycle biomarkers. PC is synthesized by LPC on the same line. The values are based on Log2FCs of biomarkers relative peak intensity compared to the control. PC, phosphatidylcholine; LPC, lysophosphatidylcholine.

Metabolic remodeling is an important manifestation of the liver’s stress response, wherein the dynamic transition between phosphatidylcholine (PC) and lysophosphatidylcholine (LPC) (Lands’ cycle) is the key regulatory pathway (Figure 5A). We found that dieting/EGCG group significantly upregulated PC, while LPC, a precursor for synthesizing PC, significantly decreased; for example, LPC (20:3) is converted to PC (20:3/18:1) by lysophosphatidylcholine acyltransferases (LPCAT) using oleoyl-CoA (18:1-CoA). We identified 12 groups of LPC to PC transformations (Figure 5B) and eight phospholipid metabolites with no corresponding relationship. The specific identification information was shown in Supplementary Table S4. As can be clearly seen from Figure 5B, the LPCs tended to decrease in the EGCG and dieting groups and further decreased in the dieting/EGCG group. We could see that the length of the carbon chain affected by liver injury ranged from 14 to 22, including both saturated and unsaturated chains, indicating that this metabolic change did not have anything to do with the length of fatty acid side chain and the number of double bonds, but was only related to the formation of PC corresponding to the addition of a fatty acid side chain on LPC. It could be seen that the changes in phospholipid component were extensive and not specific to a specific phospholipid component. The changes of these biomarkers showed that dietary restriction exacerbating liver injury induced by EGCG could cause metabolic abnormalities in Lands’ cycle.

### Dietary Restriction Exacerbating EGCG Hepatotoxicity Disrupts SM-CM Cycle

**Figure 6:**
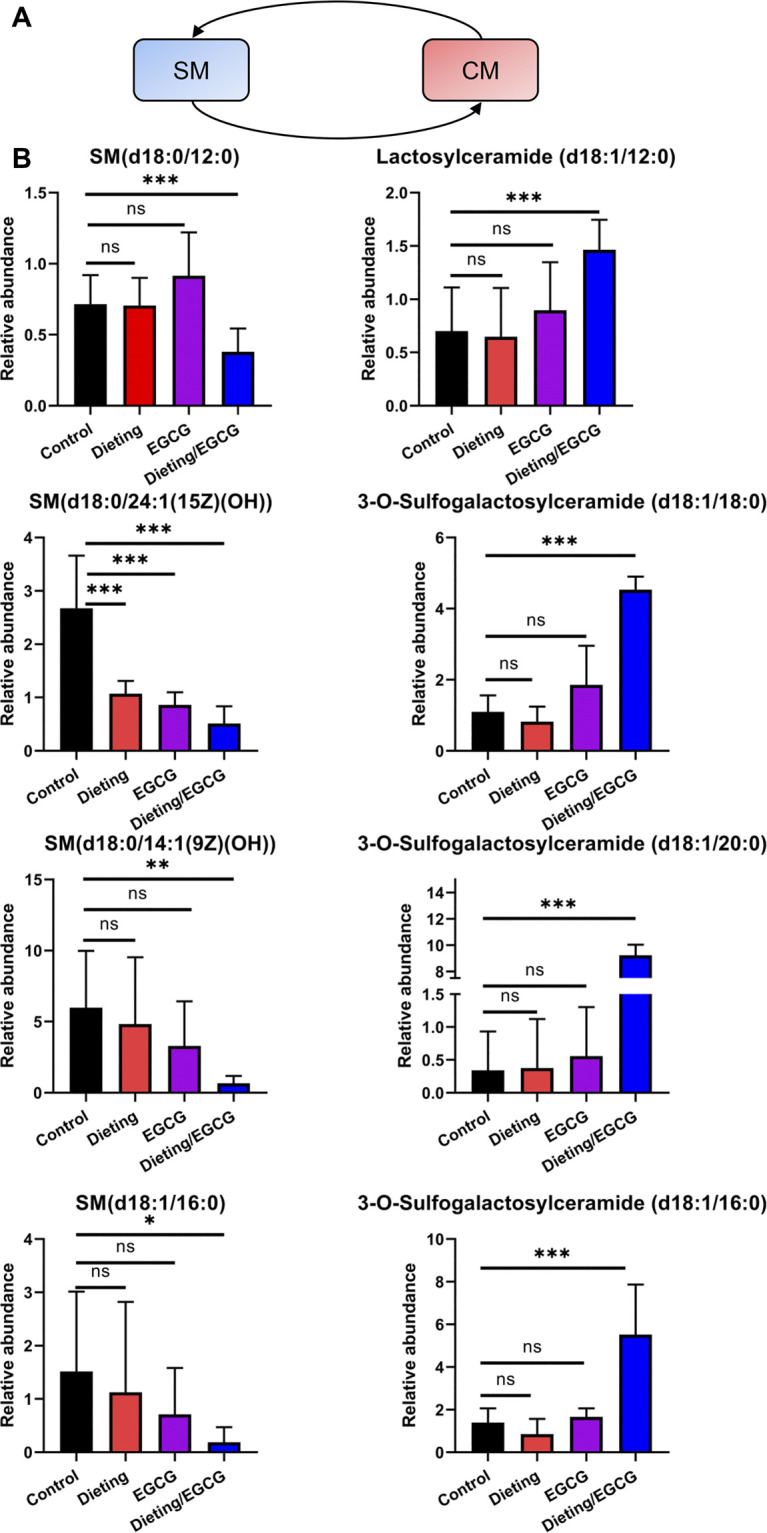
Dieting exacerbating EGCG hepatotoxicity disrupts SM-CM cycle. **(A)** SM-CM cycle. Blue represents downregulation and red represents upregulation. **(B)** Relative abundance of metabolites in SM-CM cycle. *p < 0.05, **p < 0.01, and ***p < 0.001; CM, ceramides; ns, no significance; SM, sphingomyelin.

The dynamic conversion between sphingomyelin (SM) and ceramides (CM) is another crucial regulatory pathway in the process of liver’s stress response (Figure 6A). Through metabolomics analysis, we found that the SM-CM cycle was broken during liver injury, which is manifested in the following: multiple sphingomyelins, including SM (d18:0/24:1 (15Z) (OH)), SM (d18:0/14:1 (9Z) (OH)), SM (d18:1/16:0), and SM (d18:0/12:0), were significantly downregulated in the dieting/EGCG group; at the same time, several ceramide metabolites, including lactosylceramide (d18:1/12:0), 3-O-sulfogalactosylceramide (d18:1/18:0), 3-O-sulfogalactosylceramide (d18:1/20:0), and 3-O-sulfogalactosylceramide (d18:1/16:0), were significantly upregulated in the dieting/EGCG group (Figure 6B). The specific identification information is shown in Supplementary Table S5. These results showed that the metabolic transition from SM to CM was one of the important characteristics of dieting exacerbating liver injury induced by EGCG.

### Dietary Restriction Exacerbating EGCG Hepatotoxicity Increases Taurine Metabolites and Taurine-Conjugated Bile Acids Levels

**Figure 7:**
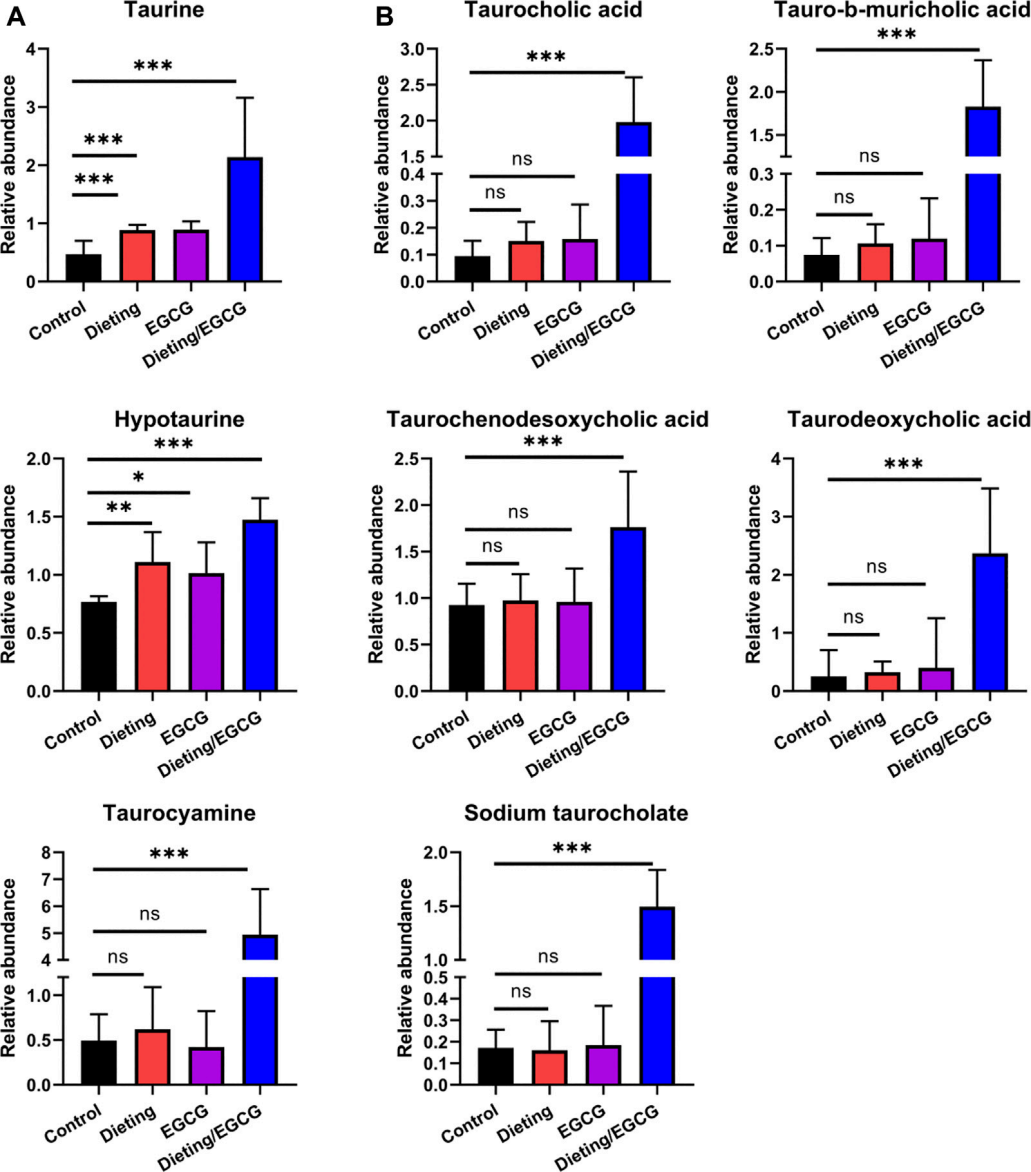
Dieting exacerbating EGCG hepatotoxicity increases taurine metabolites and taurine-conjugated bile acids. **(A)** Relative abundance of taurine, hypotaurine, taurocyamine. **(B)** Relative abundance of taurine-conjugated bile acids. *p < 0.05, **p < 0.01, and ***p < 0.001; ns, no significance.

In addition to the above metabolic characteristics, we also found that the levels of taurine and taurine-conjugated bile acids increased significantly. Taurine and its metabolites, hypotaurine and taurocyamine, were significantly higher in the dieting/EGCG group than in the dieting or EGCG group (Figure 7A). Taurine-conjugated bile acids, including taurodeoxycholic acid, tauro-b-muricholic acid, taurocholic acid, sodium taurocholate, and taurochenodesoxycholic acid, were not significantly different between the dieting and EGCG groups but increased more than three times in the dieting/EGCG group (Figure 7B). Specific identification information is shown in Supplementary Table S6.

## Discussion

With increasing obesity globally, there has been a steady increase in the consumption of GTE for weight loss. EGCG is the main component of GTE, and its big selling point is fat burning. However, the potential mechanism of such claimed weight loss effect of EGCG has not been fully characterized. Thus, we used metabolomics to reveal the effects between EGCG and dietary restriction on mice. The results showed that either EGCG treatment or dieting could regulate lipid metabolism, pentose phosphate pathway and other metabolic pathways, to inhibit fatty acid synthesis and to enhance the oxidation of fatty acid in mice. In addition, either EGCG or dietary restriction could reduce gluconic acid, indicating that pentose phosphate pathway may be inhibited, and nicotinamide adenine dinucleotide phosphate (NADPH), an important raw material for fatty acid elongation, could be reduced, which in turn would lead to the slowdown of fatty acid synthesis. These results suggested that EGCG could simulate the fatty acid consumption effect caused by dieting and had the so-called “fat-burning” effect.

However, the reports of adverse reactions related to HDS for weight loss, especially the liver injury caused by GTE, have increased significantly to a leading cause of HDS-DILI in recent years. It has been shown that a single large dose of EGCG (1,500 mg/kg) can cause liver injury in normal mice (Lambert et al., 2010). As known, drug-induced liver injury is affected by drugs, the host and the environment, of which the host’s state could be an important risk factor (Fontana, 2014). In this study, we observed for the first time that 400 mg/kg EGCG (equivalent to four times the common daily dose) could cause significant increase of transaminase and hepatocyte damage on a dietary restriction mice model; however, in normal mice, the same dose of EGCG did not cause liver injury. The above results suggested that dieting could increase the risk of EGCG-induced liver injury, with the characterization of combined toxicity potentiation. This is the first time that dieting has been proved to be a risk factor for EGCG hepatotoxicity by experimental evidence.

We further investigated the metabolic underlying mechanism wherein dietary restriction could increase the risk of EGCG-induced liver injury. We found that enhancement of fatty acid peroxidation pathway was caused by combining EGCG treatment and dietary restriction. It generated excessive oxidative pro-inflammatory lipid metabolites (9(10)-EpOME, 9,10-DHOME, 12(13)-EpOME, 12,13-DHOME) of linoleic acid. Moreover, the levels of eicosanoid oxidative metabolites (15-deoxy-delta12, 14-PGJ2, leukotriene D4, and thromboxane A2) in the downstream of arachidonic acid increased, which have been shown to promote inflammation and cell damage (Hall et al., 2017). It could be seen that liver injury caused by EGCG and dieting may be related to the increase of pro-inflammatory lipid peroxidation products, which further mediates tissue inflammation and injury. Except for the overlap of metabolic pathways regulation, other mechanisms could lead to the rising risks of EGCG administration during diet. For example, the blood concentration of EGCG increased more than 3.5-fold in the fasting condition that the EGCG exposure of liver tissue lifted in Beagle dogs, thus exacerbating the risk of liver injury (Kapetanovic et al., 2009).

We also characterized some metabolic features related to EGCG treatment plus dietary restriction-induced liver injury. Among them, the disturbances of Lands’ cycle, SM-CM cycle, and bile acids are crucial. Firstly, we observed the significant decrease of LPC and increase of its corresponding PC components in dieting/EGCG group. This feature of disturbance in Lands’ cycle has been reported as an important metabolic feature of various liver injuries such as steatohepatitis or alcoholic liver disease (Matsubara et al., 2011), which illustrated the liver injury phenotype found in our experiment. Similarly, the second metabolic feature—SM level decrease and CM level increase—reflects the disturbance of SM-CM cycle. Notably, the conversation of LPC-PC cycle and SM-CM cycle is controlled by LPCAT and sphingomyelin phosphodiesterase (SMPD, also known as sphingomyelinase), respectively. Such two key metabolic enzymes’ overexpression has been demonstrated as important features of hepatocytes in response to chemical toxin-induced liver injury (Liu et al., 2014). In addition, due to the combined inhibition of EGCG and dietary restriction on fatty acid synthase (FASN) and carnitine palmitoyl transferase-1 (CPT-1), cytoplasmic fatty acyl CoA accumulates and condenses with serine to produce 3-ketosphinganin and is then metabolized by ceramide synthetase to produce CM (Khiewkamrop et al., 2018). CM is a functional sphingolipid, and the upregulation of CM is an important signal for the activation of apoptosis cascade increasing the expression of pro-apoptotic protein Bcl2 and inducing apoptosis of various cells (Siskind et al., 2010). The high abundance of CM could promote ROS production by directly acting on mitochondrial respiratory electron chains (Abadie et al., 2001). It was indicated that increased CM may not only be a metabolomic characteristic of EGCG liver injury, but also participate in the process of liver injury. Last but not least, dysregulated taurine-conjugated bile acids are suggested to play roles in several diseases, which may be related to the high expression of bile acid transporter induced by hepatocyte injury signal.

Taken together, the disturbances of Lands’ cycle, SM-CM cycle, and taurine-conjugated bile acids may be the metabolomic characteristics of EGCG and dieting-induced liver injury, and some active metabolites may also be partially involved in the liver injury process. These metabolic characteristics could be utilized in clinical monitoring of the incidence risk of EGCG hepatotoxicity. Though this paper fully characterizes the metabolic alteration reflecting *in vivo* biological processes, further causality relationship and mechanism researches are worthy explored.

It is worth pointing out that few reports of liver injury were a result of the consumption of green tea in China, which is significantly different from the situation in the United States. Green tea is mainly used as a beverage in China, with 2–3 g of natural tea leaves soaked in water each time, where the average EGCG content is 0.7% in the natural tea beverage (James et al., 2018). However, in the United States, HDS products using GTEs are made up of complete polyphenols extracted from tea leaves with further purified EGCG or artificially added EGCG, and the EGCG content in some GTE products exceeds 90% (Hopley, 2005). The dose of tea polyphenols ingested when consuming tea is very low and obviously lower than that of GTE products in the United States, which means that we take the daily GTE product containing 800 mg of EGCG as the dosage comparable to drinking 114 g of green tea in one day. In addition, tea is traditionally consumed as a beverage in China; its extracts are not concentrated and used for weight loss, nor is it used for dieting, and people often drink tea before or after meals, so it can be explained from one aspect that green tea does not cause liver injury in China.

## Conclusion

To summarize, low dose of EGCG can increase fatty acid decomposition and inhibit fatty acid synthesis to a certain extent, which simulates the effect of dietary restriction on fatty acid metabolism and has certain fat loss effect. However, consuming EGCG while dieting could excessively disturb lipid metabolic pathways and lead to liver injury in mice, which indicates that dietary restriction could increase the risk of liver injury caused by EGCG. This happens to be a dilemma for people who plan to lose weight, as dieting and consuming EGCG concurrently may speed up weight loss, but it also leads to a higher risk of liver injury.

## Data Availability Statement

The original contributions presented in the study are included in the article/Supplementary Material. Further inquiries can be directed to the corresponding authors.

## Ethics Statement

The animal study was reviewed and approved by the Center for Laboratory Animal Welfare and Ethics of the Fifth Medical Center of the Chinese PLA General Hospital.

## Author Contributions

All authors contributed to the study conception and design. ZS, JZ, and YG wrote the manuscript. XX, YL, GZ, and JW designed this research. JZ, ZS, LZ, CT, YH, and PL conducted the research. ZS, YG, JZ, XZ, ZZ, and ZB analyzed the data. MN contributed to new reagents/analytical tools. All authors commented on previous versions of the manuscript. All authors read and approved the final manuscript.

## Funding

This work was supported by a grant from the National Natural Science Foundation of China (Grant Nos.: 81630100 and 81721002).

## Conflict of Interest

The authors declare that the research was conducted in the absence of any commercial or financial relationships that could be construed as a potential conflict of interest.
