# Supplementary material for: Epigallocatechin Gallate During Dietary Restriction — Potential Mechanisms of Enhanced Liver Injury
Source: Front Pharmacol. 2021 Jan 29;11:609378. doi: 10.3389/fphar.2020.609378 (PMC7878556; doi:10.3389/fphar.2020.609378)
Supplement: Supplementary file 1 [file datasheet1.pdf]

Supplementary Material

Chromatography and mass spectrometry conditions

The metabolic profiling analysis of the biofluids was conducted on an Agilent 6550 iFunnel Q-TOF LC/MS (Agilent Technologies, USA). The sample sequence was random and 4 μL aliquot of each sample was injected onto a ZORBOX RRHD C18 analytical column (2.1 mm i.d. × 100 mm, 1.8 μm i.d., Agilent Technologies, USA), the column temperature was maintained at 30°C. For the ESI+ analysis, separation was achieved with a 25 min linear gradient with the mobile phases of solvent A (Water spiked with 0.1% formic acid) and solvent B (Acetonitrile spiked with 0.1% formic acid). The flow rate was set as 0.30 mL/min. The gradient was used as follows: a linear gradient of 100% A over initial-1.0 min, 100–60% A over 1.0–9.0 min, 60–10% A over 9.0–19.0 min, 10–0% A over 19.0–21.0 min, 100% B over 21.0–25.0 min. The eluent was introduced to the mass spectrometer directly.

For mass spectrometry, the Agilent 6550 Q-TOF/MS with an electrospray ionization source (ESI) in both positive and negative mode was used. The electrospray source parameters were fixed as follows: electrospray capillary voltage was 3.5 kV in negative ionization mode and 4 kV in positive ionization mode. The mass range was set from m/z 50 to 1000. Gas temperature was 225°C in negative ionization mode and 225°C in positive ionization mode. Gas flow was 13 L/min. Nebulizer was set to 20 pisg (negative)and 20 pisg (positive). Sheath gas temperature was 275°C and sheath gas flow was 12 L/min. Nozzle voltage was 2000 V in both negative and positive mode. For internal mass calibration during the MS analysis, reference masses 121.0509 (Purine, $[C_5H_4N_4+H]^+$) and 922.0098 (HP-0921, $[C_{18}H_{18}O_6N_3P_3F_{24}+H]^+$) were used in positive mode, and 112.9856 (TFANH4, $[C_2H_4O_2NF_3-NH_4]^-$) and 1033.9881 TFANH4 + HP-0921, $[C_{20}H_{22}O_8N_4P_3F_{27}-NH_4]^-$) were used in negative mode.

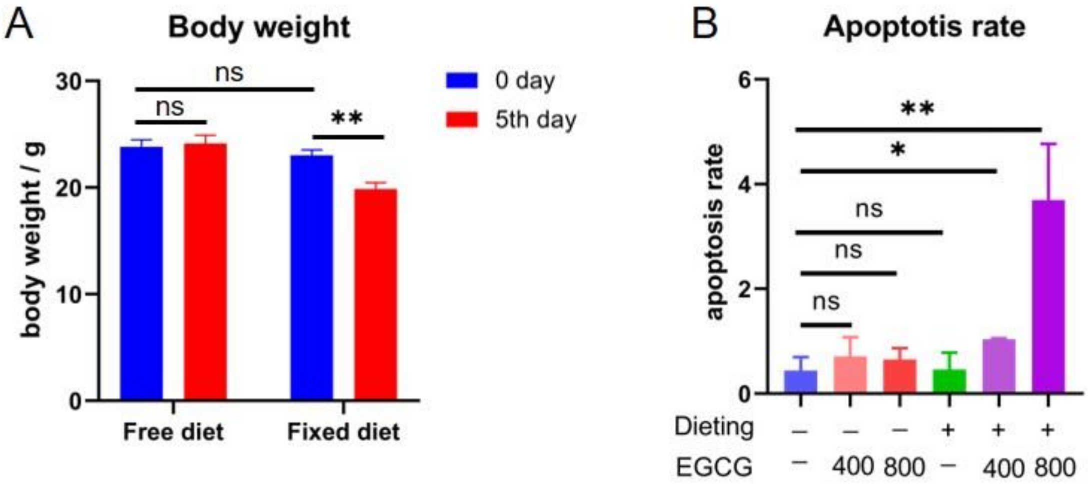

Supplementary Figure 1. (A) The average body weight of mice in the free diet and the fixed diet group after 5 days. **(B)** Apoptosis rate was performed to determine the extent of liver apoptosis.

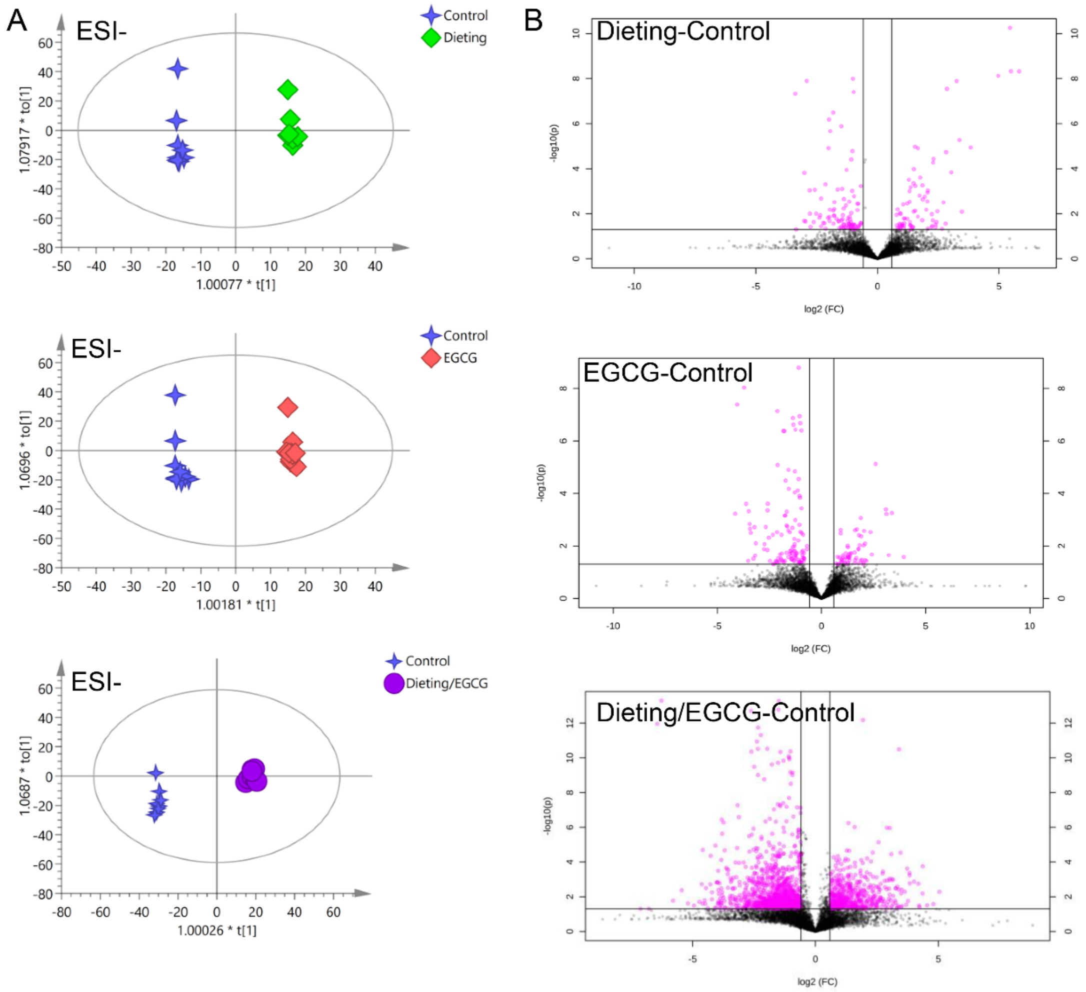

Supplementary Figure 2. Metabolomic analysis of mice plasma in negative ESI model. n=8 individuals/group. **(A)** OPLS-DA score plot in negative ESI model between Dieting, EGCG, Dieting/EGCG and Control. OPLS-DA, orthogonal projection to latent structures discriminant analysis. **(B)** Volcano plot in negative ESI model between Dieting, EGCG, Dieting/EGCG and Control. OPLS-DA, orthogonal projection to latent structures discriminant analysis.

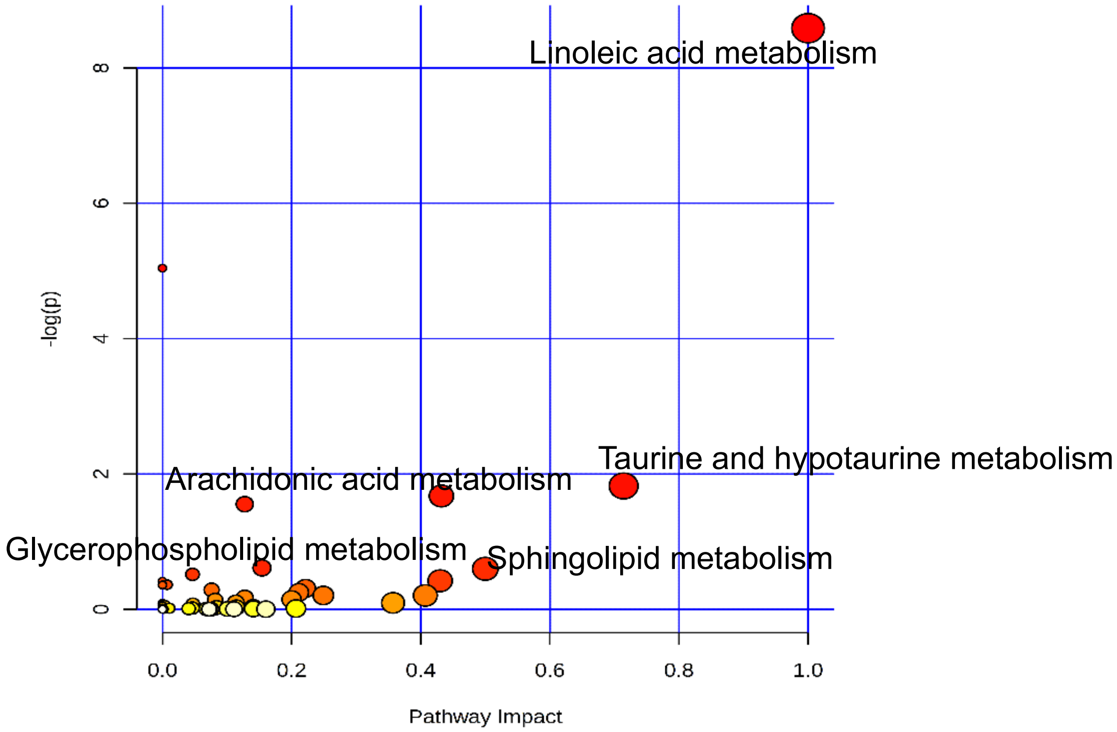

Supplementary Figure 3. Metabolic pathway analysis of liver injury from metabolites between Dieting/EGCG and Control.

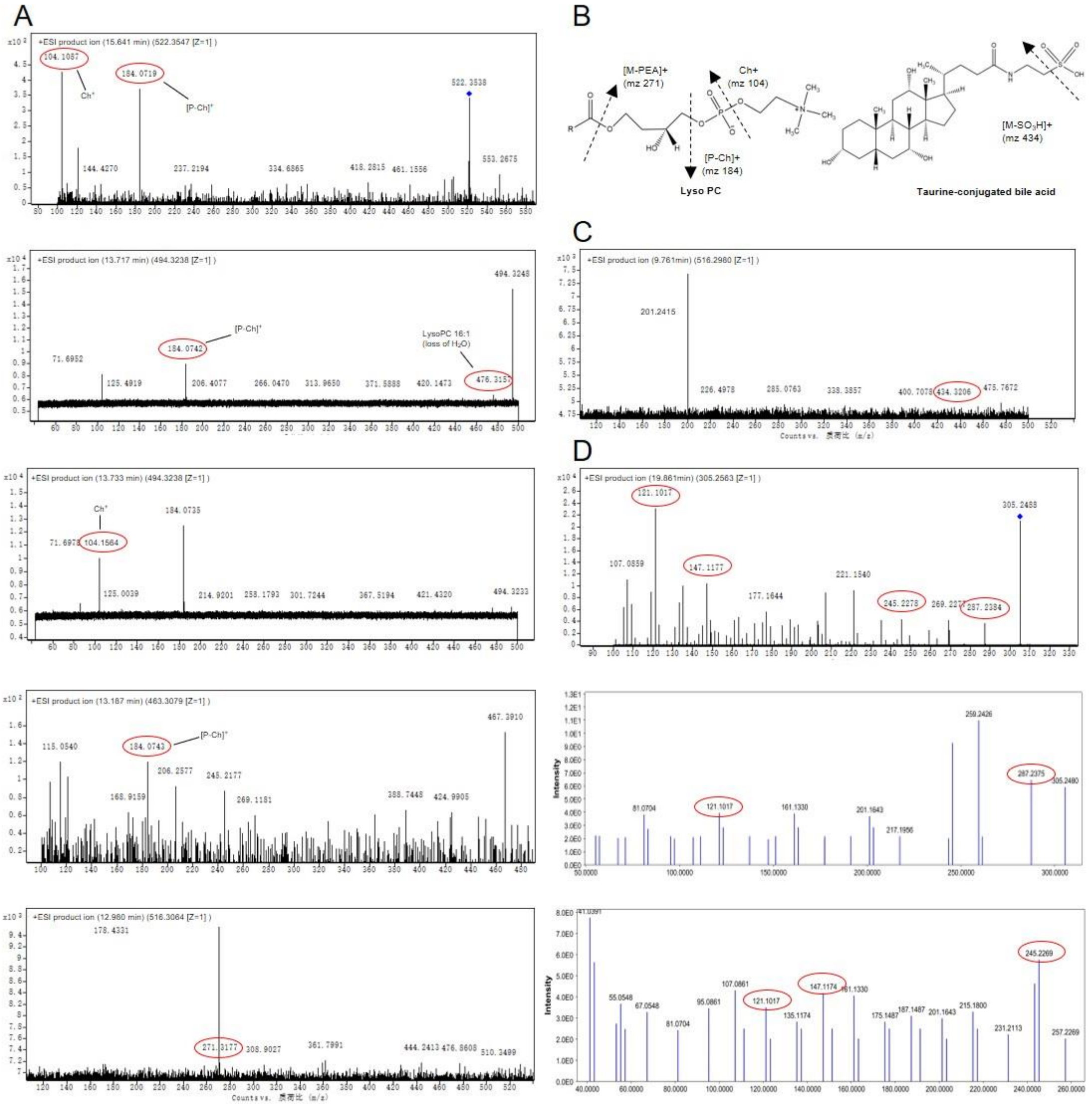

Supplementary Figure 4. Detection of representative metabolisms in the positive ion mode. (A) MS/MS spectrum of main LysoPCs and PCs. (B) Fragmentation patterns for LysoPCs and Taurine-conjugated bile acids. (C) MS/MS spectrum of the taurine-conjugated bile acid. (D) MS/MS spectrum of arachidonate and its standard spectrum.

Table S1 Results of shared metabolites between EGCG and Dieting group.

Number	Scan mode	MZ	RT	Candidate	FC	P	FC	P	Δppm
					Dieting vs Control		EGCG vs Control		
1	ESI-	195.0503	0.83	Gluconic acid	0.61	1.22E-05	0.25	0.0500	4
2	ESI-	241.1805	12.24	3-oxo-tetradecanoic acid	3.70	0.0049	2.80	0.0042	2
3	ESI-	281.2477	15.31	Oleic Acid	0.33	0.0009	0.49	1.27E-05	3
4	ESI+	131.1078	10.28	Heptanoic acid	0.13	0.0007	0.24	0.0001	8
5	ESI+	155.1048	8.41	(E)-2,6-Dimethyl-2,5-heptadienoic acid	0.42	8.192E-08	0.24	0.0004	12
6	ESI+	573.3845	11.60	Vitamin D2 3-glucuronide	0.50	1.05E-06	0.53	2.48E-05	10

Table S2 Identification of significantly changed metabolites in Arachidonate metabolism.

Number	Scan mode	MZ	RT	Candidate	FC<br>Dieting/EGCG vs Control	P	VIP	p(corr)	Δppm
1	ESI+	305.2563	19.56	Arachidonate	0.12	0.0000	1.35	0.81	29
2	ESI+	317.2201	10.44	15-Deoxy-Delta12,14-PGJ2	1.78	0.0087	0.77	-0.51	28
3	ESI+	497.2756	8.31	Leukotriene D4	3.08	0.0073	0.54	-0.50	15
4	ESI-	351.2271	12.92	Thromboxane A2	2.18	0.0012	0.63	0.56	27

Table S3 Identification of significantly changed metabolites in Linoleate metabolism.

Number	Scan mode	MZ	RT	Candidate	FC<br>Dieting/EGCG vs Control	P	VIP	p(corr)	Δppm
1	ESI-	279.2325	18.99	Linoleate	0.56	1.25E-05	0.61	-0.73	2
2	ESI+	297.2402	16.46	9(10)-EpOME	1.74	0.0025	0.48	-0.56	7
3	ESI+	297.2407	17.39	12(13)-EpOME	2.00	0.0794	0.53	-0.34	7
4	ESI-	313.2375	13.78	9,10-DHOME	5.23	0.0094	0.86	0.53	3
5	ESI-	313.2375	13.83	12,13-DHOME	4.24	0.0170	0.80	0.49	3

Table S4 Identification of significantly changed metabolites in Lands' cycle.

Number	Scan mode	MZ	RT	Candidate	FC<br>Dieting/EGCG vs Control	P	VIP	p(corr)	Δppm
1	ESI+	468.3079	13.02	LysoPC(14:0)	0.64	3.17E-06	0.62	0.77	1
2	ESI+	482.3240	16.78	LysoPC(15:0)	0.49	5.74E-13	0.82	0.94	0
3	ESI+	494.3238	13.57	LysoPC(16:1(9Z))	0.30	1.86E-12	1.20	0.93	1
4	ESI-	522.3542	17.97	LysoPC(18:0/0:0)	0.44	4.53E-07	0.93	-0.79	3
5	ESI+	516.3064	13.61	LysoPC(18:4(6Z,9Z,12Z,15Z))	0.53	0.0062161	0.69	0.52	4
6	ESI+	550.3861	17.34	LysoPC(20:1(11Z))	0.35	1.32E-13	1.13	0.94	1
7	ESI+	548.3675	16.14	LysoPC(20:2(11Z,14Z))	0.60	0.003944	0.52	0.53	6
8	ESI+	546.3543	14.87	LysoPC(20:3(8Z,11Z,14Z))	0.14	8.05E-17	1.32	0.97	2
9	ESI+	544.3381	17.06	LysoPC(20:4(8Z,11Z,14Z,17Z))	0.23	8.57E-08	0.88	0.84	3
10	ESI+	572.3685	17.34	LysoPC(22:4(7Z,10Z,13Z,16Z))	0.34	2.22E-13	1.15	0.94	4
11	ESI+	570.3531	15.63	LysoPC(22:5(7Z,10Z,13Z,16Z,19Z))	0.31	2.07E-10	1.04	0.90	4
12	ESI+	568.3386	15.30	LysoPC(22:6(4Z,7Z,10Z,13Z,16Z,19Z))	0.11	0.0002	4.12	0.67	2
13	ESI+	782.5693	18.15	PC(14:0/22:4(7Z,10Z,13Z,16Z))	2.38	0.0003	0.71	-0.64	0
14	ESI+	794.5731	20.12	PC(15:0/22:5(7Z,10Z,13Z,16Z,19Z))	2.11	0.0002	0.73	-0.65	4
15	ESI+	808.5819	19.63	PC(16:1(9Z)/22:4(7Z,10Z,13Z,16Z))	3.12	0.0004	1.14	-0.64	4
16	ESI+	746.5949	18.94	PC(18:0/P-16:0)	3.32	0.0009	1.07	-0.60	14
17	ESI-	738.5328	20.01	PC(18:4(6Z,9Z,12Z,15Z)/P-16:0)	3.55	0.0053	1.08	0.50	15
18	ESI+	810.5984	12.81	PC(20:1(11Z)/18:3(6Z,9Z,12Z))	2.56	0.0045	0.77	-0.53	3
19	ESI+	772.5799	16.49	PC(20:2(11Z,14Z)/15:0)	1.60	0.0008	0.50	-0.59	7
20	ESI+	794.5996	18.11	PC(20:3(8Z,11Z,14Z)/P-18:1(9Z))	3.64	0.0002	1.07	-0.66	8
21	ESI+	802.5409	19.71	PC(20:4(5Z,8Z,11Z,14Z,17Z)/18:4(9Z,12Z,15Z))	8.21	0.0056	1.23	-0.52	3

22	ESI+	796.5916	19.27	PC(22:4(7Z,10Z,13Z,16Z)/15:0)	3.26	0.0007	0.82	-0.61	8
23	ESI+	828.5476	11.17	PC(22:5(7Z,10Z,13Z,16Z,19Z)/18:4(6Z,9Z,12Z,15Z))	3.38	0.0023	0.78	-0.56	7
24	ESI+	804.5518	19.38	PC(22:6(4Z,7Z,10Z,13Z,16Z,19Z)/16:1(9Z))	2.56	0.0031	1.26	-0.54	2
25	ESI+	552.4020	19.30	LysoPC(20:0)	0.14	1.23E-11	1.95	0.92	1
26	ESI+	522.3547	16.42	LysoPC(18:1(9Z)/0:0)	0.37	3.67E-08	0.99	0.85	1
27	ESI+	833.5887	20.43	PC(20:4(5Z,8Z,11Z,14Z)/20:3(8Z,11Z,14Z))	2.44	0.0014	0.72	-0.58	5
28	ESI+	806.5692	16.67	PC(22:6(4Z,7Z,10Z,13Z,16Z,19Z)/16:0)	1.93	7.74E-06	0.69	-0.74	0
29	ESI+	766.5426	17.03	PC(20:5(5Z,8Z,11Z,14Z,17Z)/15:0)	2.13	0.0038	0.66	-0.55	6
30	ESI+	854.5667	20.24	PC(22:6(4Z,7Z,10Z,13Z,16Z,19Z)/20:4(8Z,11Z,14Z,17Z))	1.89	0.0008	0.64	-0.60	3

[1]The underlined compounds were not listed as heatmap in Lands' cycle

[2]PC, Phosphatidylcholine. LPC, Lyso Phosphatidylcholine.

Table S5 Identification of significantly changed metabolites in SM-CM cycle.

Number	Scan mode	MZ	RT	Candidate	FC Dieting/EGCG vs Control	P	VIP	p(corr)	Δppm
1	ESI+	305.2023	19.43	SM(d18:0/24:1(15Z)(OH))	0.35	0.0015	0.94	0.52	29
2	ESI+	689.5384	7.63	SM(d18:0/14:1(9Z)(OH))	0.31	0.0058	1.35	0.54	23
3	ESI+	703.5738	20.20	SM(d18:1/16:0)	0.13	0.0037	1.19	0.51	2
4	ESI-	649.5260	3.08	SM(d18:0/12:0)	0.08	0.0004	0.80	0.51	5
5	ESI+	806.5692	16.67	Lactosylceramide (d18:1/12:0)	1.93	0.0011	0.69	-0.74	0
6	ESI+	808.5819	19.63	3-O-Sulfogalactosylceramide (d18:1/18:0)	3.12	0.0000	1.14	-0.64	4
7	ESI+	836.6097	16.70	3-O-Sulfogalactosylceramide (d18:1/20:0)	5.04	0.0004	1.511	-0.54	8
8	ESI+	780.5502	8.79	3-O-Sulfogalactosylceramide (d18:1/16:0)	2.76	0.0005	0.80	0.51	27

[1]SM, sphingomyelin. CM, ceramides.

Table S6 Identification of significantly changed metabolites in taurine metabolism and taurinated bile acids.

Number	Scan mode	MZ	RT	Candidate	FC Dieting/EGCG vs Control	P	VIP	p(corr)	Δppm
1	ESI+	110.0274	0.85	Hypotaurine	1.56	0.0005	0.50	-0.63	3
2	ESI-	124.0071	0.92	Taurine	1.87	0.0091	0.56	0.50	3
3	ESI+	168.0461	11.14	Taurocyamine	5.19	0.0004	0.48	0.51	4
4	ESI-	498.2882	9.33	Taurodeoxycholic acid	7.13	0.0028	0.92	0.58	3
5	ESI-	514.2831	9.38	Tauro-b-muricholic acid	14.30	0.0017	0.77	0.56	4
6	ESI+	516.2980	9.14	Taurocholic acid	11.47	0.0013	0.75	-0.59	14
7	ESI+	538.2827	7.91	Sodium taurocholate	4.96	0.0041	1.00	-0.70	1
8	ESI+	367.2574	17.32	Taurochenodesoxycholic acid	1.42	0.0500	0.60	-0.52	6
